# The SWEDEGRAFT Saphenous Vein Trial: A Study of No-Touch *vs.* Nordic Grafts?

**DOI:** 10.21470/1678-9741-2025-0195

**Published:** 2026-09-10

**Authors:** Michael R Dashwood

The recent SWEDEGRAFT Trial on no-touch (NT) saphenous vein grafts (SVG) published in the European Heart Journal was based on data from a total of 902 coronary artery bypass grafting (CABG) patients operated in eight centers in Sweden and one in Denmark^[^1^]^. The proportion of patients with occluded/stenosed SVGs in 454 patients receiving NT grafts was compared with 446 patients receiving conventional (CT) grafts. The incidence of leg wound complications was also recorded. At 3.5-year followup, 90/454 (19.8%) NT and 107/446 (24.0%) CT SVGs were occluded with the authors concluding that “No-touch vein graft harvesting for CABG was not superior to conventional open harvesting”^[^1^]^. This result is in complete contrast to many other trials including that described by Samano et al. showing a superior long-term patency of NT grafts at up to a 16-year follow-up^[^2^]^.

A number of matters arise questioning the reliablility of the SWEDEGRAFT trial. For example, in their Editorial, “The SWEDEGRAFT trial: when absence of evidence is not evidence of absence”, Gaudino and Sandner raise a number of critical issues relating to this study^[^3^]^. Firstly, the use of outdated vein graft failure data which “represents a critical flaw in sample size calculation”. Secondly, the low rate of only 78% of patients undergoing follow-up computed tomography angiography to assess graft patency is considerably lower than the 96% follow-up rate reported in the recent trial on a larger cohort by Tian et al.^[^4^]^. Extrapolating results from 22% of non-imaged to the imaged patients seriously limits the robustness of the SWEDEGRAFT patency results.

Superior NT SVG patency is due to the preservation of normal saphenous vein (SV) architecture that is achieved when the surrounding cushion of perivascular fat (PVF) remains intact. Handling the NT SV by the PVF prevents spasm and avoids the distensionand surgery-induced damage that is caused at CT harvesting^[^5^]^. In the early years, when the NT technique was under development, routine samples of NT and CT SV were collected for histological examination. These studies showed that normal SV architecture was maintained and that endothelial integrity, an intact vasa vasorum and preservation of PVF-derived factors was achieved in NT grafts whereas these structures were damaged or removed when using CT SV for CABG (Figure 1)^[^5^]^. As yet, no such data has been presented for the NT and CT SVGs used in the SWEDEGRAFT trial.

**Fig. 1:**
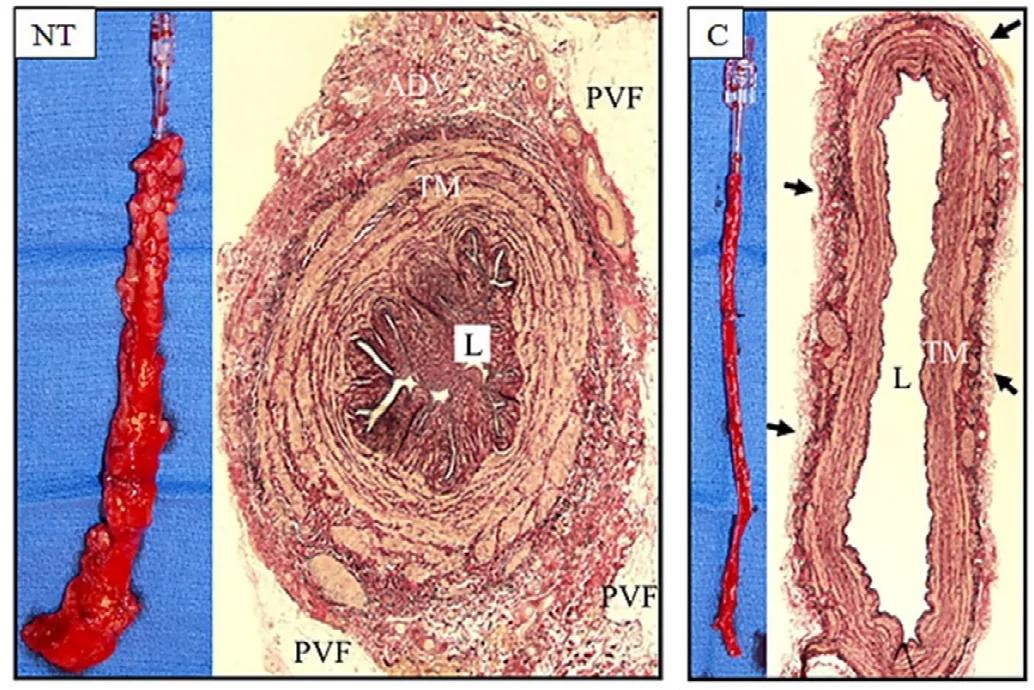
Examples of no-touch (NT) and conventional (C) saphenous vein (SV) grafts. The left panel shows a representative NT explant with surrounding cushion of fat intact. The transverse section of NT SV shows an intact surrounding cushion of perivascular fat (PVF), adventitia (ADV), and thick media (TM). As this vessel has not been distended, the lumen (L) is thrown into folds. The right panel shows a representative C SV explant separated from its PVF. The transverse section of C SV exhibits various forms of damage. The PVF has been removed and much of the ADV has been damaged almost to the level of the external elastic lamina (small arrows). The TM is thinner than that of the NT SV, and the L is dilated and regions of endothelium denuded due to high-pressure intraluminal distension. (From: Samano et al.^[^5^]^).

The SWEDEGRAFT data was based on occlusion rates of 90/454 of NT and 107/446 CT patients (*P* = 0.15) at a mean follow-up time of 3.5 years, where Thelin et al. suggest that this study “…..proves the high quality of cardiac surgery in the Nordic countries”. This seems a bold statement, particularly given the positive NT SVG results of many other studies^[^2^]^ including that described recently by Tian et al.^[^4^]^ In this study, vein graft occlusion was assessed by computed tomography angiography on more than double the number of patients than the SWEDEGRAFT trial. At mean follow-up of three years, the NT group showed a significantly lower vein graft occlusion rate of 105/1140 (9.2%) than the CT group 152/1141 (13.3%) (*P* = 0.002). Furthermore, there was a lower incidence of recurrent angina in patients receiving NT than CT grafts (6.2% *vs.* 8.4%; *P*=0.03; respectively). The superiority of the NT technique is further supported by a number of meta-analyses including that performed recently on data from seven studies by Deng et al.^[^6^]^ Here, a total of 3334 patients and 5798 SVGs were used where, at mean angiographic follow-up at 11.6 months, SVG per graft and per patient occlusion rates were significantly lower in the NT compared with the CT group (*P* < 0.001 for both groups).

Considering the number of trials and meta-analyses showing the superiority of the NT technique, the question is whether all surgeons participating in the SWEDEGRAFT trial harvested SVGs according to the established protocol? For example, was preoperative ultrasound mapping performed to assess SV quality and was any form of distension used? Was the required amount of PVF preserved on SVs? How were SVs stored during CABG: in heparinised blood or antispastic solutions? For CT SV was distension used and was pressure measured? Did any PVF remain intact? Apart from the SVG explants illustrated in the Structured Graphical Abstract of the SWEDEGRAFT publication, no other examples are provided. These particular examples are from Orebro patients and similar to those shown in the review by Samano et al.^[^5^]^ As no examples are provided other than those from Orebro, where the NT technique was introduced, we are unable to assess the ‘high quality’ of the SVGs used by individual Nordic surgeons and/or other participating centers. There is some discussion in the SWEDEGRAFT study regarding the issue of wound healing where the NT technique “was associated to a higher risk of early and late leg wound complications”. Again, this may be due to a lack of surgeons’ experience in the NT technique. The recent study by Tian et al.^[^4^]^ not only shows a significant reduction in NT SVG occlusion but this group has previously stated that there is “a continuous decline in the rates of major leg wound complications” in NT patients through “practice and dedicated training”. Presumably most surgeons participating in the SWEDEGRAFT study had more previous experience in the CT than the NT technique. It is therefore possible that the conflicting results of the SWEDEGRAFT compared with other studies is explained by data obtained by Nordic surgeons using ‘good’ CT and ‘bad’ NT harvesting.

## Artificial Intelligence Usage

The author declares that no artificial intelligence tool was used in the preparation of this article.
